# Countermeasures to oppose Alberta's use of the notwithstanding clause to exclude transgender athletes from women's sport

**DOI:** 10.3389/fspor.2026.1772828

**Published:** 2026-02-19

**Authors:** Marcus Mazzucco

**Affiliations:** Faculty of Kinesiology and Physical Education, University of Toronto, Toronto, ON, Canada

**Keywords:** discrimination, inclusion, sports law, sports policy, strategic litigation, transgender

## Abstract

In Alberta, Canada, the provincial government has enacted legislation to ban transgender women and girls from participating in women's sport. The government invoked the notwithstanding clause in the *Canadian Charter of Rights and Freedoms* to shield the ban from legal challenge for violating the right to non-discrimination, among other constitutional rights. Various countermeasures are available to the Canadian sport system to oppose Alberta's ban and circumvent its use of the notwithstanding clause. These countermeasures involve constitutional and corporate laws, the federal government's spending powers, privacy law, and administrative law. Sport organizations, the federal government, and athletes can pursue these countermeasures to ensure that affected transgender women and girls are not unfairly denied the opportunity to participate in women's sport.

## 1 Introduction

In 2024, the Government of Alberta in Canada enacted the *Fairness and Safety in Sport Act* (the “*Act*”) (1). The *Act* requires the boards of certain sport organizations in the province to adopt and implement policies respecting “fairness and safety” in sport, in accordance with the requirements set out in the *Act* and its regulation. The preamble to the *Act* emphasizes the physical and mental health benefits of sport, as well as the government's commitment to promote fairness, safety, and broad-based participation in sport (1).

At first glance, the statutory scheme seems innocuous and even well-intentioned. However, the government's communications about the legislation reveal its plan to use the *Act* to ban transgender (trans) women and girls from participating in women's sports due to the perceived threat they pose to the integrity of the women's competition category (2). The regulation made under the *Act*, which came into force on September 1, 2025, confirms this intent and provides a clearer picture of how the legislation operates (3).

Rather than defer to the boards of sport organizations to develop eligibility policies regulating the participation of trans athletes in women's sport, the legislation requires sport organizations to adopt policies that exclude from the women's competition category any athlete aged 12 years or older whose sex at birth is not female (3). Sport organizations must ensure compliance with this eligibility requirement in two ways. First, when an athlete registers to participate in women's sport, the relevant sport organization must require the athlete (or their parent/guardian, in the case of a minor) to confirm in writing that the athlete was assigned the female sex at birth (3). Second, if the athlete's eligibility to participate in women's sport is challenged by a third party, the sport organization must direct the athlete (or their parent/guardian) to provide a copy of the athlete's birth registration document as proof of their sex at birth (3).^1^

The *Act* also requires sport organizations to annually notify the Alberta Minister of Tourism and Sport about requests for, and the establishment of, any mixed-gender or mixed-sex competition categories (1, 3). While the government has described this reporting requirement as supporting the creation of opportunities for trans women and girls to participate in sport (8), it is notable that the legislation does not require the boards of sport organizations to take proactive steps to establish such mixed-gender or mixed-sex competition categories. This passive approach to creating opportunities for inclusive sport stands in stark contrast to the mandatory obligation imposed on sport organizations to exclude trans women and girls from women's sport. Further, the government's focus on reporting requirements suggests that it may wish to monitor whether sport organizations attempt to avoid the operation of the legislation by converting women's-only categories into mixed-gender or mixed-sex categories.

The *Act* has received criticism by those within and outside the Canadian sport system, including federal government officials [e.g., (9)], national sport organizations [see (10)], universities [e.g., (11)], associations of medical and legal professionals [e.g., (12, 13)], and human rights advocacy groups [e.g., (14)].

As other scholars have noted, the *Act* is discriminatory and unsupported by scientific evidence, will impose burdens on sport organizations, and will cause significant harms to athletes in its implementation, including depriving trans women and girls from the health and social benefits of sport (15–17).

On December 11, 2025, the Alberta government amended the *Act* to specify that it operates notwithstanding the rights in the *Canadian Charter of Rights and Freedoms* (the “*Charter*”),^2^ the *Alberta Bill of Rights*, and the *Alberta Human Rights Act* (19). These human rights instruments prohibit discrimination on the basis of sex, gender, gender expression, and gender identity.^3^

The use of the notwithstanding clause to override the rights in the *Charter* in the context of sport is unprecedented in Canada but follows a growing trend amongst provincial governments to use the clause to achieve policy goals at all costs (24). Arguably, this defensive step is evidence of the government's understanding and acceptance that the *Act* violates various human rights.

Alberta's invocation of the notwithstanding clause will stop any lawsuits that seek to challenge the *Act* for violating certain human rights protections.^4^ Yet, it will not completely shield the *Act* from opposition. Several countermeasures exist to challenge the implementation of the legislation based on constitutional and corporate laws, the spending powers of the federal government, privacy law, and administrative law.

The remainder of this article examines these countermeasures from the perspectives of various policy actors in the Canadian sport system – specifically, sport organizations, the federal government, and athletes.

## 2 Policy options and their implications

### 2.1 Sport organizations

The *Act* only applies to certain sport organizations – specifically, public and private elementary, secondary and post-secondary schools, school boards, and provincial sport organizations (PSOs) (1).^5^ The selection of these specific organizations is tied to the Government's legislative authority under Canada's *Constitution Act, 1867*, which divides law-making powers between the federal and provincial governments (26).

The province does not have exclusive law-making jurisdiction to regulate sport and can only do so to the extent that the aspect being regulated falls within the province's legislative competence. For example, section 93 of the *Constitution Act, 1867* gives provincial governments exclusive authority to make laws in relation to education (26). As a result, schools and school boards in Alberta are provincially incorporated and subject to Alberta laws respecting their operation,^6^ including in matters relating to sport.

In contrast, Alberta does not have legislative authority to regulate all aspects of PSOs.^7^ Rather, it has legislative authority to regulate them as provincially incorporated entities as a matter of corporate law (26). This scope of authority is reflected in the *Act's* definitions (which, define a PSO as an entity incorporated under Alberta's not-for-profit corporation legislation) and in the obligations the *Act* imposes on the boards of PSOs to establish and implement eligibility policies (1). Accordingly, the *Act* does not regulate the corporate affairs of organizations incorporated federally or in another province or territory. The Government of Alberta has conceded this by acknowledging that the *Act* will not apply to federally incorporated national sport organizations (NSOs) or PSOs in other provinces that visit Alberta for a competition (30).

Therefore, PSOs in Alberta may be able to avoid the application of the *Act* by incorporating themselves federally under the *Canada Not-for-profit Corporations Act* (31). Alternatively, PSOs in Alberta can amalgamate with their NSOs, or PSOs outside of Alberta, to form a new corporation that is incorporated federally or in another province. Such vertical and horizontal integration of sport organizations was recently recommended by the Future of Sport in Canada Commission as a way to strengthen alignment and create efficiencies in the sport system (32).

### 2.2 Federal government

The Government of Canada can use its spending powers to counteract the exclusionary effects of Alberta's legislation and promote inclusive sport.

For example, if an Alberta PSO pursued either of the options described above (i.e., federal incorporation or amalgamation with an entity outside of Alberta), then the PSO could become ineligible to receive financial grants from the Government of Alberta, which could jeopardize the PSO's ability to operate. However, the Government of Canada can offset this financial loss by providing grants to the newly constituted or amalgamated Alberta PSO, as the federal government's spending power is not confined to national-level sport (33).

The Government of Canada could also adopt a framework on inclusion inspired by the Statement on Trans and Gender-Diverse Inclusion in Sport issued by former federal Minister of Sport Carla Qualtrough (9) and make compliance with this framework a requirement for federally funded sport organizations, including NSOs. The framework could require NSOs to ensure that their eligibility rules, and the eligibility rules of their PSO members, start from a place of inclusion and only restrict the eligibility of trans athletes in women's sport based on peer-reviewed, scientific evidence relevant to a sport. Such a policy framework could also encourage NSOs to stop hosting sport competitions in Alberta as a way of applying pressure on the Government of Alberta to reconsider its legislation [see (34)].

Finally, the Government of Canada could support the implementation of this new policy framework by funding research into the regulation of gender in sport, similar to the scientific review conducted in 2022 that was commissioned by Sport Integrity Canada (formerly, known as the Canadian Centre for Ethics in Sport) and funded by the Government of Canada (35).

### 2.3 Athletes

Athletes can circumvent Alberta's use of the notwithstanding clause by using legal tactics outside of the *Charter*, the *Alberta Bill of Rights*, and the *Alberta Human Rights Act*.

For example, privacy laws have been recognized as a means to challenge the regulation of gender in sport [see (36, 37)], as illustrated by a recent privacy complaint filed in Canada against the World Anti-Doping Agency for its role in allowing doping data to be used to exclude intersex and trans athletes from women's sport at the international level (38).

Alberta's *Personal Information Protection Act* (“*PIPA*”) applies to the collection, use, and disclosure of personal information by PSOs and private schools when they carry out commercial activities (39).^8^ *PIPA* defines “commercial activity” as any transaction, act or conduct, or any regular course of conduct, that is of a commercial character, such as the selling of a membership or services (39).^9^ It is arguable that a PSO's or a private school's collection of the birth sex information of an athlete for the purpose of registering them as a member of a women's-only sport team or as a competitor in a women's-only sport event for a fee constitutes a commercial activity within the meaning of *PIPA* [cf. (41, 42)].

Under *PIPA*, an organization can only collect, use, or disclose personal information for purposes that are reasonable – that is, for purposes a reasonable person would consider appropriate in the circumstances (39). An athlete could file a complaint with Alberta's Information and Privacy Commissioner alleging that a PSO's or a private school's collection and use of birth sex information to determine the eligibility of an athlete for the women's competition category is not for a reasonable purpose due to the lack of scientific evidence justifying a blanket exclusion of trans athletes from the women's category [cf. (43)]. If the complaint is upheld and, as a result, the PSO or the private school is prohibited from collecting and using the birth sex information, then they would not be able to exclude trans women and girls from the women's competition category under the terms of their eligibility policy.

Finally, athletes can challenge the lawfulness of the regulation made under the *Act* by bringing an application for judicial review to the Alberta Court of King's Bench. The Supreme Court of Canada recently held in the case of *Auer v. Auer* that courts reviewing the validity of a regulation must assess whether it is consistent with the purpose of its parent statute using a reasonableness standard of review (44). Prior to this decision, Canadian courts applied a more deferential standard when reviewing the validity of a regulation and would determine a regulation to be invalid only if it was “irrelevant, extraneous, or completely unrelated” to the purpose of its enabling statute [(45), para. 28].

A strong argument can be made that the regulation made under the *Act* is inconsistent with the purpose of the *Act* for two reasons. First, the *Act* describes a scheme whereby the board of a sport organization will have some discretion to establish and implement its own eligibility policies for women's sport. While the *Act* states that such policies need to comply with the regulation, the *Act* also states that the Minister of Tourism and Sport may establish non-binding guidelines respecting the eligibility policies, which suggests that the Legislature wanted to preserve some autonomy for sport organizations. However, the regulation fetters any such autonomy and discretion by strictly prescribing rules for the content and implementation of eligibility policies.

Second, the regulation is inconsistent with the purpose of the *Act*. As noted above, the purpose of the *Act*, as set out in its preamble, is to ensure broad-based participation in sport based on the principles of safety and fairness. A regulation that requires sport organizations to adopt a blanket ban on the participation of trans women and girls in women's sport, without any peer-reviewed, scientific evidence to justify the ban [see (35)] and without providing any alternative opportunities for trans athletes to participate in sport, is inconsistent with the purpose of the *Act*.

## 3 Actionable recommendations

Based on the policy options and implications examined in the previous section, the following actionable countermeasures are recommended for various policy actors in the Canadian sport system.

PSOs in Alberta should consider incorporating themselves under federal legislation or amalgamating with their NSOs or PSOs outside of Alberta to avoid the operation of the *Act*.
The Government of Canada should use its spending power in the following ways:
  Fund any PSOs in Alberta that lose access to provincial funding due to their incorporation under federal legislation or amalgamation with other entities;
  Adopt a Framework on Trans and Gender-Diverse Inclusion in Sport that requires federally funded NSOs to ensure that their eligibility rules, and the eligibility rules of their PSO members, start from a place of inclusion and only restrict the eligibility of trans athletes in women's sport based on peer-reviewed, scientific evidence relevant to a sport; and
  Fund peer-reviewed, scientific research into the regulation of gender in sport.
Athletes should file complaints with the Information and Privacy Commissioner of Alberta alleging that the collection and use of birth sex information by a PSO or a private school is not for a reasonable purpose, contrary to *PIPA*.
Athletes should commence an application for judicial review in the Alberta Court of King's Bench to challenge the validity of the regulation made under the Act on the basis that it is inconsistent with the purpose of the *Act*.

## 4 Conclusion

The Government of Alberta has enacted legislation to ban trans women and girls from women's sport, and in an unprecedented move has invoked the notwithstanding clause to shield the ban from legal challenge under certain human rights laws. Various countermeasures are available to the Canadian sport system to oppose Alberta's legislative efforts and ensure that trans women and girls are not unfairly denied the opportunity to participate in women's sport. These countermeasures involve constitutional and corporate laws, the federal government's spending powers, privacy law, and administrative law, and can be pursued by various policy actors, including sport organizations, the federal government, and athletes.

## Footnotes

^1^A birth registration document is different than a birth certificate. A birth registration document is the originating document used to register a birth under Alberta's *Vital Statistics Act*, and it is maintained by Alberta's Registrar of Vital Statistics (4). An individual who is at least 12 years old or their parent/guardian may apply to the Registrar for a certified copy of the birth registration document by providing proof of their age and identity and paying a fee of $20 (5, 6). The Registrar can refuse to provide the certified copy if they believe it will be used for an unlawful or improper purpose, or if they believe that providing the copy is not in the public interest (4). However, the application form for requesting a copy of the birth registration document does not require the applicant to disclose the reason for their request (7). As a result, it is unclear how the Registrar could exercise their statutory discretion to deny providing a copy of the document.

^2^Specifically, the rights in sections 2 and 7 to 15 of the *Charter*, which guarantee freedom of expression, freedom of association, the right to life, liberty and security of the person, the right to privacy (including informational privacy), and the right to non-discrimination, among other rights (18).

^3^See *Alberta Bill of Rights* at section 1(1) (20), *Alberta Human Rights Act* at section 4 (21), and the *Charter* at section 15 (18), which was interpreted by the Quebec Superior Court in *Centre for Gender Advocacy v. Attorney General of Quebec* (22) as including gender identity as an analogous ground protected from discrimination, a finding which was endorsed by the Supreme Court of Canada in *Hansman v. Neufeld* (23).

^4^For example, the notwithstanding clause was used to shield from legal challenge a related Alberta law that prohibits the provision of gender-affirming care to minors, and this use resulted in the removal of a court injunction that had suspended the operation of the law while its constitutionality was being litigated (25).

^5^The *Act* also applies to organizations or classes of organizations that may be prescribed by regulation (1). At the time this article was submitted for publication, there are no other organizations or classes of organizations prescribed by regulation that are subject to the *Act*. If the Alberta Government were to prescribe additional organizations to be subject to the *Act*, it would be confined to those organizations that fall within its legislative authority to regulate, as discussed in this section.

^6^See *Education Act* (27) and *Post-secondary Learning Act* (28).

^7^PSOs are members of national sport organizations (NSOs) and are responsible for overseeing and administering their sport at the provincial level, in accordance with their own rules and the rules of their NSO (29).

^8^In contrast, public universities and school boards (and by extension public elementary and secondary schools) are “public authorities” subject to Alberta's *Protection of Privacy Act* (40).

^9^The definition of “commercial activity” also includes the operation of a private elementary, secondary and post-secondary school (39).
